# Sabre tibia in Paget’s disease of bone

**DOI:** 10.1093/rap/rkad032

**Published:** 2023-03-25

**Authors:** Kristina E N Clark, Ali S M Jawad

**Affiliations:** Department of Rheumatology, Barts Health NHS Trust, London, UK; Department of Rheumatology, Barts Health NHS Trust, London, UK

A 67-year-old man presented with long-standing painful deformity of the left leg. Originally from Ghana, he had been living in the UK for 19 years. He had no past medical history. Physical examination revealed a prominent sabre-like convexity of the tibia (Fig. 1A). Radiographs showed cortical thickening, trabecular enlargement and bowing of the tibia (Fig. 1B). SPECT CT showed increased activity in the proximal two-thirds of the tibia only (Fig. 1C). Serum alkaline phosphatase level was raised, at 206 U/l (normal range 45–129 U/l). The diagnosis was Paget’s disease of bone. A single dose of zoledronic acid was given i.v., with subsequent improvement in pain and normalized serum alkaline phosphatase within 6 months.

**Figure 1.**
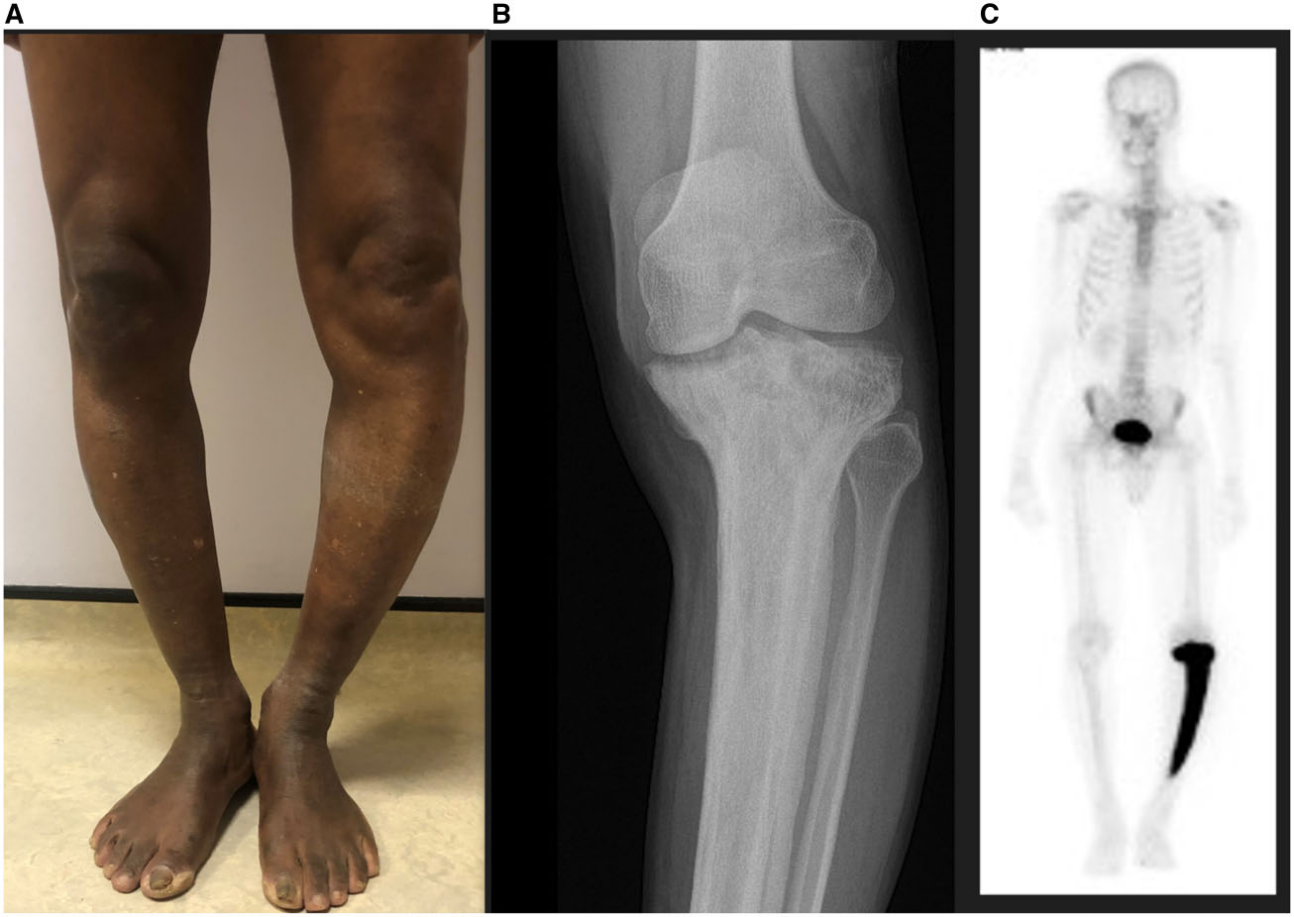
Clinical images and radiographic depiction of sabre tibia in Paget’s disease. (A) Prominent sabre-like convexity of the right leg. (B) Radiograph (anteroposterior view) showin cortical thickening, trabecular enlargement and bowing of the tibia. (C) SPECT CT showed increased activity in the proximal two-thirds of the tibia

Sabre shin or sabre tibia in adults is mainly attributable to Paget’s disease of bone causing cortical thickening and trabecular enlargement, leading to lateral bowing or convexity of the tibia [1]. Sabre tibia in children is caused by congenital syphilis and rickets; the malformation is usually in the form of sharp anterior rather than lateral bowing and usually bilateral. Other features of congenital syphilis, such as Hutchinson’s teeth and mulberry molars, are present. Malformation of the tibia, caused by periosteitis, has also been reported in acquired syphilis, but the deformity is not very impressive [2]. It is important to think of Paget’s disease within the differential of painful deformities, even in ethnic minorities where the incidence is low, because it responds extremely well to zoledronic acid i.v.

## Data Availability

The data underlying this article are available in the article.
